# Remodeling Tumor Immune Microenvironment by Using Polymer-Lipid-Manganese Dioxide Nanoparticles with Radiation Therapy to Boost Immune Response of Castration-Resistant Prostate Cancer

**DOI:** 10.34133/research.0247

**Published:** 2023-10-03

**Authors:** Abdulmottaleb E. Zetrini, HoYin Lip, Azhar Z. Abbasi, Ibrahim Alradwan, Taksim Ahmed, Chunsheng He, Jeffrey T. Henderson, Andrew M. Rauth, Xiao Yu Wu

**Affiliations:** ^1^ Advanced Pharmaceutics and Drug Delivery Laboratory, Leslie Dan Faculty of Pharmacy, University of Toronto, M5S 3M2, Toronto, ON, Canada.; ^2^ Departments of Medical Biophysics and Radiation Oncology, University of Toronto, M5G 1L7, Toronto, ON, Canada.

## Abstract

Despite substantial progress in the treatment of castration-resistant prostate cancer (CRPC), including radiation therapy and immunotherapy alone or in combination, the response to treatment remains poor due to the hypoxic and immunosuppressive nature of the tumor microenvironment. Herein, we exploited the bioreactivity of novel polymer–lipid manganese dioxide nanoparticles (PLMDs) to remodel the tumor immune microenvironment (TIME) by increasing the local oxygen levels and extracellular pH and enhancing radiation-induced immunogenic cell death. This study demonstrated that PLMD treatment sensitized hypoxic human and murine CRPC cells to radiation, significantly increasing radiation-induced DNA double-strand breaks and ultimately cell death, which enhanced the secretion of damage-associated molecular patterns, attributable to the induction of autophagy and endoplasmic reticulum stress. Reoxygenation via PLMDs also polarized hypoxic murine RAW264.7 macrophages toward the M1 phenotype, enhancing tumor necrosis factor alpha release, and thus reducing the viability of murine CRPC TRAMP-C2 cells. In a syngeneic TRAMP-C2 tumor model, intravenous injection of PLMDs suppressed, while radiation alone enhanced recruitment of regulatory T cells and myeloid-derived suppressor cells. Pretreatment with PLMDs followed by radiation down-regulated programmed death-ligand 1 and promoted the infiltration of antitumor CD8^+^ T cells and M1 macrophages to tumor sites. Taken together, TIME modulation by PLMDs plus radiation profoundly delayed tumor growth and prolonged median survival compared with radiation alone. These results suggest that PLMDs plus radiation is a promising treatment modality for improving therapeutic efficacy in radioresistant and immunosuppressive solid tumors.

## Introduction

Radiation therapy (RT), as a monotherapy or in combination with surgery and androgen deprivation, is a standard treatment for localized and advanced prostate cancer (PCa) [1,2]. The primary anticancer effect of radiation is cell death following induction of DNA double-strand breaks [3]. If such injury occurs at sufficient levels, the DNA damage can induce P53-mediated cell killing, resulting in overt cellular destruction through apoptosis, macroautophagy, and cell necroptosis [4]. In particular, the latter is associated with the release of damage-associated molecular patterns (DAMPs) and activation of stimulator of interferon genes [5]. Such effects ultimately result in the stimulation of local dedritic cells that specialize in the presentation of new tumor-associated antigens to targets such CD8^+^ T cells, promoting antitumor immunity and tumor growth inhibition [5]. Radiation treatment can also induce immunogenic cell death (ICD), characterized by the expression of DAMPs, including translocation of “eat me” signaling calreticulin (CRT) to the plasma membrane [6] and secretion of “find me” signals, e.g., adenosine triphosphate (ATP) [7] and high mobility group box 1 (HMGB1) into the extracellular space [8]. Induction of endoplasmic reticulum (ER) stress and autophagy have also been shown to facilitate the release of DAMPs [9–11]. The released DAMPs are powerful immunological triggers that stimulate the recruitment of dendritic cells and cytotoxic T cells into tumor, inducing an antitumor response [7].

Despite this process, tumor-infiltrating T cells face a highly hostile tumor microenvironment (TME) characterized by hypoxia, acidosis, excess reactive oxygen species (ROS), immunosuppressive cells, and localized up-regulation of programmed death-1 and programmed death-ligand 1 (PD-L1) [12]. These features within the TME act to suppress tumor-killing functions of antitumor immune cells, negatively influencing the therapeutic outcomes of many conventional anticancer treatments, including RT [13–15]. For example, tumor hypoxia can promote the infiltration of immature myeloid cells and facilitate their conversion to highly immunosuppressive myeloid-derived suppressor cells (MDSCs), causing immune evasion [16–18]. Recruitment of immunosuppressive cells to the tumor is associated with enhanced tumor growth and poor treatment outcomes in patients with cancer [19]. Acidosis, attributed to the increased production of lactate due to hypoxia-modified glycolysis, has also been found to suppress the tumor immune response [20]. Consistent with this, the function of antitumor effectors such as T cells has been shown to be impaired, undergoing a state of anergy and apoptosis at a low-pH environment [21]. Dysregulation of ROS production in cancer cells is another factor that has been shown to significantly alter the TME and inhibit antitumor immunity [22]. Specifically, expression of PD-L1 has been found to be regulated by ROS [22]. Consistent with this, treatment of cells with ROS-inducing agents, such as buthionine sulfoximine and paclitaxel, has been shown to increase the expression of PD-L1 on the surface of tumor-associated macrophages, subsequently promoting tumor growth [22].

Given these factors, immunomodulation of RT has previously been employed to overcome the suppression of adaptive immune responses by tumor cells and their associated microenvironment [23,24]. In addition, immune checkpoint inhibitors such as antibodies targeting cytotoxic-T-lymphocyte-associated protein 4 or PD1/PD-L1 have led significant clinical improvement in patients with some cancer types such as melanoma [25]. However, castration-resistant prostate cancer (CRPC) does not respond to immune checkpoint inhibitors [26,27], attributable in part to the mutations in DNA damage proteins, the composition and density of immune cells in the tumor, the hostile TME conditions, and low expression of immune checkpoint proteins such as PD-L1 [28]. Therefore, appropriate treatment modalities for PCa are yet to be developed [29]. Given the pleiotropic effects of RT and the pathophysiological properties of the TME that can lead to immunosuppression, simultaneous modulation of these factors may represent an innovative strategy to enhance the antitumor effects of RT. Recently, our laboratory developed clinically applicable polymer–lipid manganese dioxide nanoparticle (PLMD) formulations and tested their ability to modulate the TME in both breast and PCa tumor models [30–34]. These PLMDs act to lower ROS levels by interacting with hydrogen peroxide, a product of tumor metabolism, reducing tumor acidity, and generating oxygen as a by-product [30]. We observed that intravenous injection of PLMDs resulted in a significant decrease in tumor hypoxia and oxidative stress in human prostate tumor xenografts [34]. We also found that the combination of PLMDs and RT significantly improved the lifetime expectancy of tumor-bearing mice, using 2 different human PCa tumor models, compared with RT alone [34]. Encouraged by these results, in the present study, we further examined the mechanism of PLMD effects with RT in modulating the tumor immune microenvironment (TIME). Human PC3 and DU145 cells, murine TRAMP-C2 cells, and tumor xenografts were used for these studies. In vitro examination revealed that the combination of PLMDs with RT increased the secretion of the ICD markers associated with induction of autophagy and ER stress. Combination treatment with PLMDs and RT also significantly reduced the expression of PD-L1 compared to RT alone in PC3 and TRAMP-C2 cells and tumors in vitro and in vivo. As shown in a syngeneic mouse model with TRAMP-C2 tumors grown in C57BL/6J mice, PLMDs plus RT increased the recruitment of antitumor immune cells, CD8^+^ T cells, and M1 macrophages while lowering the percentage of immunosuppressive cells, regulatory T cells (Tregs), and MDSCs within the tumor, resulting in increased apoptosis, slowed tumor growth, and prolonged host survival.

## Results

### PLMDs amplify RT-induced DNA damage and ICD biomarkers under hypoxic conditions

**Fig. 1.**
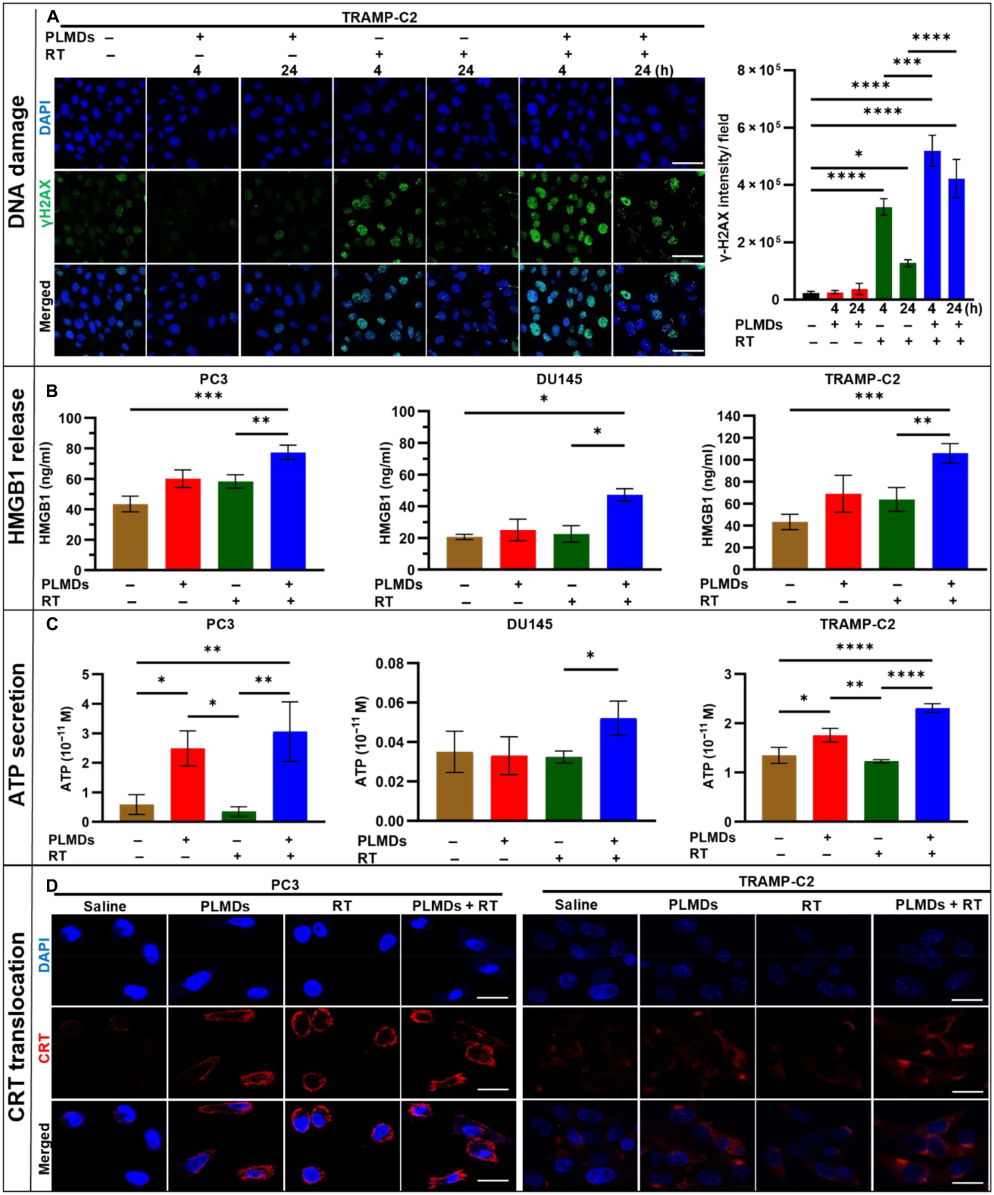
PLMDs plus RT enhanced DNA damage and induced ICD markers in vitro. (A) Immunofluorescence images of γ-H2AX staining of TRAMP-C2 cells 4 and 24 h after treatment with saline, 12.5 μM PLMDs, RT (6 Gy), and PLMDs + RT under hypoxic conditions. Scale bars, 50 μm. (B) HMGB1 release and (C) ATP secretion in hypoxic PC3, DU145, and TRAMP-C2 cells in vitro. (D) Representative CLSM images of CRT exposure in hypoxic PC3 and TRAMP-C2 cells in vitro. Scale bars, 20 μm. PCa cells were treated with saline, PLMDs (12.5 μM), RT (6 Gy), or PLMDs plus RT under hypoxic conditions. HMGB1 release and ATP release were assessed 24 h post-treatment, and CRT exposure was assessed 4 h post-treatment. Results mean +SD for ≥ 3 independent experiments. *P* < 0.05 is considered statistically significant. *P* < 0.05 (*), *P* < 0.005 (**), *P* < 0.0005 (***), and *P* < 0.0001 (****). DAPI, 4′,6-diamidino-2-phenylindole.

Given the well-characterized role of DNA damage in antitumor immune cell induction [35] and the role of hypoxia in reducing radiation-induced DNA damage, the effect of PLMDs on RT-induced DNA damage was investigated in hypoxic TRAMP-C2 cells. Following treatment with either saline, PLMDs (12.5 μM), RT (6 Gy), or PLMDs plus RT for 4 or 24 h, relative levels of phosphorylated form of H2A histone family member X (γ-H2AX), a marker of DNA double-strand breaks, were determined by immunofluorescence using confocal laser scanning microscopy (CLSM) (Fig. 1A). The doses of PLMDs and RT used in this study were selected on the basis of our previous study [34]. As shown in Fig 1A, PLMDs alone had no significant effect on the expression of γ-H2AX compared to saline. RT alone significantly increased the expression of γ-H2AX foci at 4 h by 14-fold compared to that in saline; however, the foci diminished dramatically by 24 h post-treatment, consistent with the expected levels of DNA repair. In contrast, pretreatment with PLMDs for 1 h followed by RT enhanced γ-H2AX expression by 22.9- and 18.6-fold compared to saline treatment at 4 and 24 h post-treatment, respectively, and by 1.6- and 3.3-fold, respectively, compared to RT alone (Fig. 1A). Strikingly, γ-H2AX expression at 24 h post-treatment did not decrease as much after PLMDs + RT treatment compared to RT treatment alone, suggesting that PLMDs sustained the DNA-damaging effects of RT (Fig. 1A). The pretreatment effect of PLMDs on hypoxic TRAMP-C2 cells may thus reflect the added extent and fixation of DNA damage induced by local oxygen generation [30,32,34].

ICD induction is the first step in the development of an antitumor response [36]. To investigate whether PLMDs alone or in combination with RT induced ICD under hypoxic conditions, we measured the development and secretion of DAMPs in hypoxic PC3, DU145, and TRAMP-C2 cells. To mimic the PCa TME, where the oxygen availability ranges from 0.3% to 1.2%, all in vitro experiments were performed at 1% O_2_ [37]. The release of HMGB1 and ATP was assayed after 24 h of treatment of hypoxic cells with saline, PLMDs (12.5 μM), RT (6 Gy), or PLMDs plus RT. PLMDs or RT alone modestly increased HMGB1 release compared to saline in PC3 and TRAMP-C2 cells and showed little effect on DU145 cells, whereas PLMDs + RT significantly enhanced HMGB1 release in all 3 cell lines examined (Fig. 1B). With respect to ATP, RT alone did not cause ATP secretion in any of the cell lines examined, whereas PLMDs exerted different effects in different cell lines. PLMDs alone significantly induced ATP secretion compared to saline in PC3 and TRAMP-C2 cells but not in DU145 cells. Similarly, PLMDs significantly increased ATP release compared to RT alone in PC3 and TRAMP-C2 cells but not in DU145 cells. Similar to HMGB1, PLMDs + RT treatment significantly elevated ATP secretion compared to RT alone in PC3, DU145, and TRAMP-C2 cells (Fig. 1C). Surprisingly, the combination treatment also led to a significant increase in ATP release compared with RT alone in DU145 cells (Fig. 1C). Notably, ATP release in saline-treated PC3 and TRAMP-C2 cells was approximately 35-fold higher than that in DU145 cells (Fig. 1C).

CRT translocation within the plasma membrane was examined using CLSM. While translocation of CRT was not observed in any saline-treated cell lines, PLMDs treatment alone or in combination with RT increased the surface exposure of CRT in both PC3 and TRAMP-C2 cells (Fig. 1D), but not in DU145 cells (Fig. S1). RT alone caused CRT translocation in PC3 cells but not in TRAMP-C2 cells (Fig. 1D). These results suggest that these 3 cell lines respond differently to PLMDs and RT treatment, potentially suggesting different molecular mechanisms of cell death.

### PLMDs alone and in combination with RT induce autophagy and ER stress in murine and human PCa cells

**Fig. 2.**
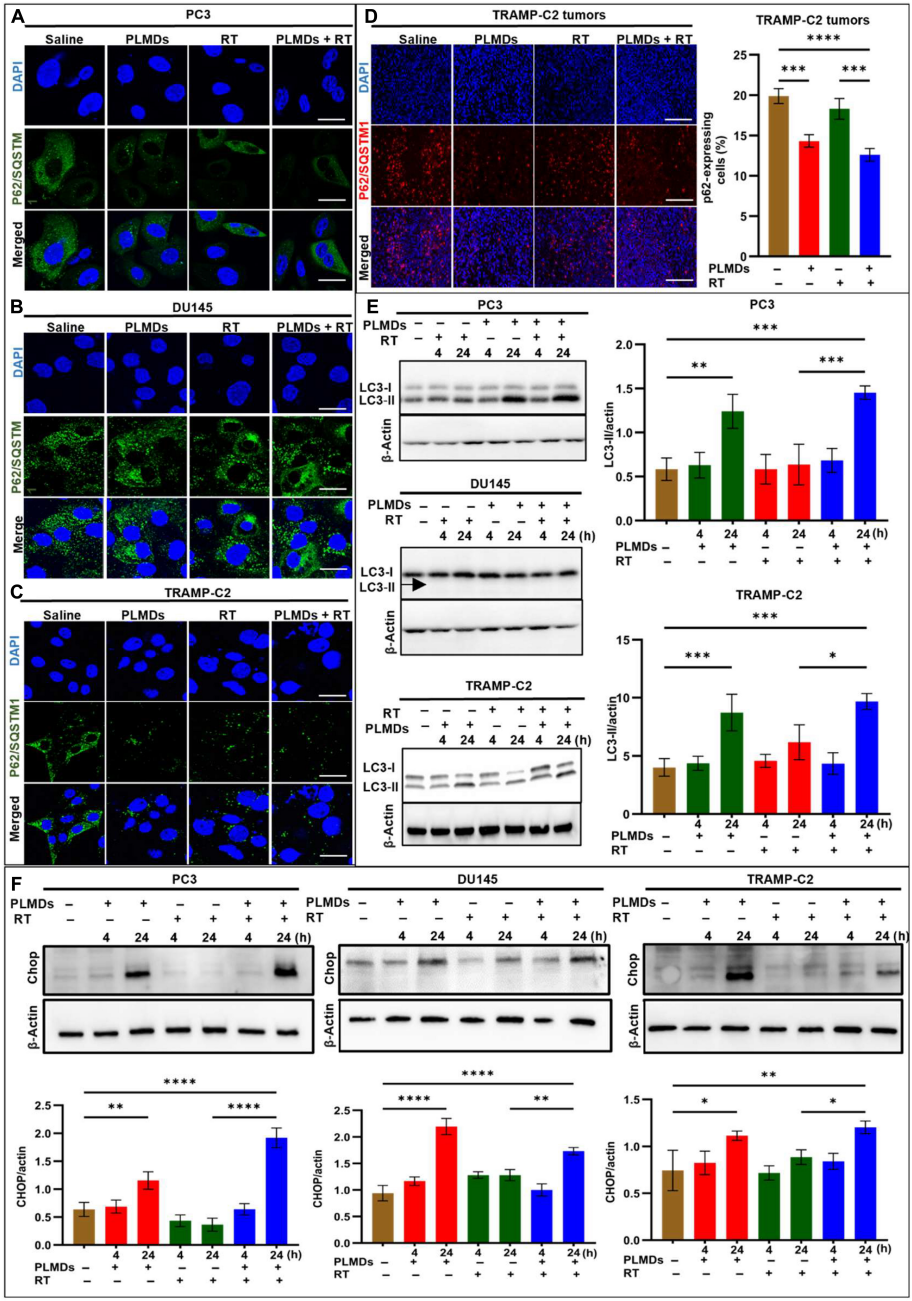
PLMDs induced autophagy and ER stress in human and murine PCa cell lines. CLSM images demonstrating the effects of saline, PLMDs (12.5 μM), RT (6 Gy), or PLMDs plus RT on autophagy biomarker, P62/SQSTM1 expression in (A) PC3, (B) DU145, and (C) TRAMP-C2 cells under hypoxic conditions in vitro. Images were captured 24 h post-treatment. Scale bars, 10 μm. (D) Immunofluorescence staining of P62/SQSTM1 in TRAMP-C2 tumors at day 2 post IV, treatment with saline, PLMDs, RT, or PLMDs plus RT. Scale bars, 200 μm. (E and F) Immunoblots of autophagy-related biomarker, LC3-I and LC3-II, and ER stress biomarker, CHOP, respectively in PC3, TRAMP-C2, and DU145 cells and the quantification relative to β-actin standard. Hypoxic PC3, DU145, and TRAMP-C2 cells were treated as mentioned above, and cell lysates were collected at 4 and 24 h post-treatment. Results are the means and SD of 3 independent experiments. *P* < 0.05 (*), *P* < 0.005 (**), *P* < 0.0005 (***), and *P* < 0.0001 (****).

The observed differences in ICD biomarkers among the different cell lines prompted us to further examine the mechanism of ICD associated with autophagy and ER stress, which are known to be required for ATP secretion, HMGB1 release, and CRT translocation [10,38].Degradation of p62/sequestosome 1(SQSTM1) and conversion of microtubule-associated protein 1A/1B-light chain 3 (LC3) from LC3-I to LC3-II, required during the induction of autophagy [39] were examined by CLSM and Western blotting. P62/SQSTM1 degradation was investigated in vitro in all 3 cell lines under hypoxic conditions and ex vivo in TRAMP-C2 tumors (Fig. 2).

In PC3 and TRAMP-C2 cells, PLMDs (12.5 μM), either alone or in combination with RT (6 Gy) reduced P62/SQSTM1 staining compared to saline (Fig. 2A and C), whereas in DU145 cells P62/SQSTM1 levels remained high regardless of the treatment (Fig. 2B). In the TRAMP-C2 tumor model, PLMDs alone or PLMDs plus RT caused significantly lower p62 levels compared to the saline or RT groups, indicating that autophagy was induced in TRAMP-C2 tumors by PLMDs-containing treatments (Fig. 2D). Immunoblotting of LC3 forms in the cell lysates in vitro revealed that the autophagic flux significantly increased in PC3 and TRAMP-C2 cells at 24 h post-treatment with PLMDs, either alone or in combination with RT, as indicated by the elevated LC3-II levels compared to saline controls. (Fig. 2E). At 4 and 24 h post-RT alone, no significant effect on the conversion of LC3-I to LC3-II was observed compared with saline. However, in DU145 cells, none of the treatments affected the conversion of LC3-I to LC3-II, as evidenced by the lack of LC3-II (Fig. 2E). These results suggest that PLMDs promote autophagy in autophagy-competent PC3 and TRAMP-C2 cells, but not in DU145 cells. ER stress was further examined in vitro via immunoblotting for the CHOP biomarker. In hypoxic cells, PLMDs alone or in combination with RT increased the expression of CHOP by 2- to 4-fold compared with saline and RT alone groups at 24 h but not at 4 h post-treatment, whereas RT alone had little effect on the ER stress response (Fig. 2F).

### PLMDs polarize hypoxic RAW264.7 macrophages toward M1 phenotype reducing TRAMP-C2 cell viability

**Fig. 3.**
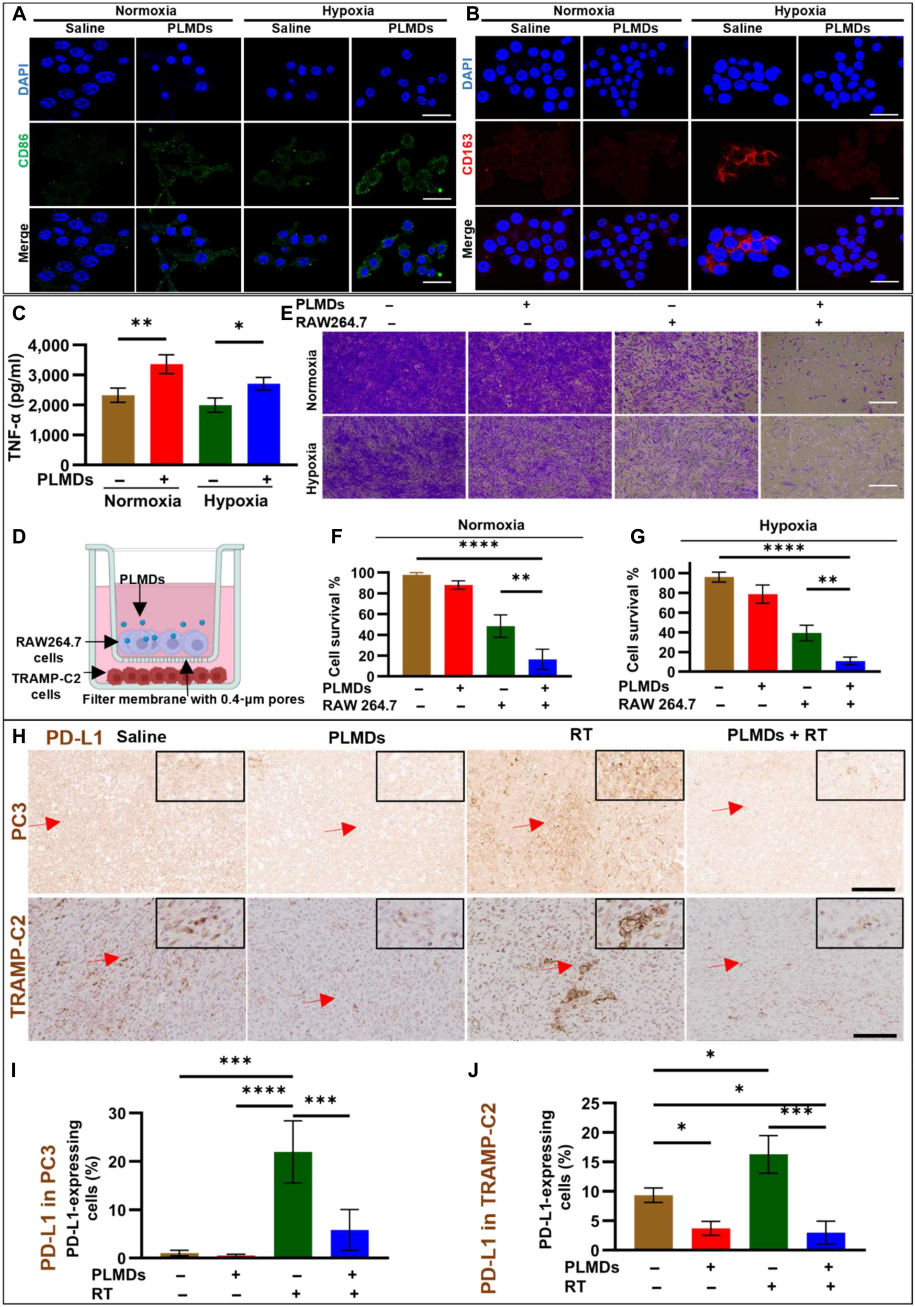
PLMDs induced M1 macrophage polarization in normoxic and hypoxic RAW264.7 macrophages in vitro and reduced the expression of PD-L1 in PC3 and TRAMP-C2 tumors in vivo. Immunofluorescence images of (A) CD86 (M1 macrophage marker), and (B) M2 macrophages marker, CD163, in RAW264.7 cells. Scale bars, 50 μm. (C) TNF-α release in the supernatant of RAW264.7 cells treated with 12.5 μM PLMDs for 24 h under normoxic or hypoxic conditions. (D) Illustration of Transwell assay for coculturing study as explained in the materials and methods. (E) Microscope images of viable TRAMP-C2 cells stained with crystal violet in the presence and absence of RAW264.7 cells or PLMDs. Scale bars, 100 μm. (F and G) Survival of TRAMP-C2 cells cocultured with or without RAW264.7 cells and/or PLMD treatment. (H) IHC images for the ex-vivo PD-L1 expression 5 d after saline, PLMDs (0.87 mg MnO_2_/kg bw), RT alone (10 Gy), and PLMDs + RT treatment in PC3 and TRAMP-C2 tumors. Scale bars, 200 μm. (I and J) Corresponding quantification of the percentages of PD-L1-expressing cells after various in vivo treatments. Saline *n* = 3, PLMDs alone *n* = 4, RT alone *n* = 4, PLMDs + RT *n* = 5. Data are expressed as means ± SD. *P* < 0.05 (*), *P* < 0.005 (**), *P* < 0.0005 (***), and *P* < 0.0001 (****).

Hypoxia and chronic ROS production have previously been shown to promote macrophage polarization toward an M2 phenotype, promoting tumor progression, metastasis, and angiogenesis [40]. Previously, we showed that PLMD treatment produced oxygen through consumption of ROS (H_2_O_2_), leading to reduced hypoxia [30,34]. Therefore, in this study we examined the effects of the ROS scavenging and oxygen generating function of PLMDs on the polarization of normoxic and hypoxic RAW264.7 macrophages by investigating the expression of M1 and M2 biomarkers. Under both normoxic and hypoxic conditions, PLMD treatment up-regulated expression of the M1 macrophage marker CD86 in RAW264.7 macrophages compared to saline controls (Fig. 3A). Under hypoxic conditions, PLMD treatment reduced the expression of M2 macrophage biomarker CD163 in RAW264.7 cells compared with saline-treated cells, while exhibiting no effect on the expression of the CD163 under normoxic conditions (Fig. 3B).

Macrophage M1 polarization is also associated with increased secretion of tumor necrosis factor-α (TNF-α), which exhibits tumoricidal effects [41]. To further investigate the impact of PLMDs on M1 polarization, TNF-α release was measured in cell supernatants under normoxic or hypoxic conditions. As shown in Fig 3C, secretion of TNF-α by RAW264.7 macrophages was significantly enhanced by PLMD treatment compared to the saline controls under hypoxic or normoxic conditions. Finally, the cytotoxicity of M1-polarized RAW264.7 macrophages against TRAMP-C2 cells was evaluated functionally in a coculture assay using a Transwell apparatus, as shown in Fig. 3D. Under both normoxic and hypoxic conditions, TRAMP-C2 cell survival was significantly reduced when RAW264.7 macrophages were treated with PLMDs compared to saline groups (Fig. 3E to G). These results demonstrate that PLMD treatment significantly potentiated the cell killing effect of RAW264.7 macrophages on TRAMP-C2 cells.

### PLMDs down-regulate PD-L1 expression when used alone or in combination with RT in human and murine PCa cells in vitro or in vivo in tumors

Programmed death-1/PD-L1 up-regulation in tumor cells and immune cells contributes to tumor immune evasion by inhibiting the antitumor effects of cytotoxic T lymphocytes (CTLs) [42]. Hypoxia and radiation induce PD-L1 overexpression in cancer cells [42–44]. Given the previous finding from our previous work that PLMDs down-regulated hypoxia-inducible factor 1α in PCa cells [34], we investigated the effect of PLMDs on down-regulating PD-L1 expression in all cell lines. Cells were grown under hypoxic conditions for 48 h and treated with either saline or PLMDs (12.5 μM MnO_2_) 1 h before irradiation (6 Gy). At 24 h post-treatment, PD-L1 expression was evaluated using CLSM. These images demonstrate that each of the cell lines examined express low levels of PD-L1 protein in saline- or PLMDs-treated groups; while radiation induced the expression of PD-L1, PLMDs plus RT did not induce PD-L1 expression (Fig. S2).

In PC3 and TRAMP-C2 prostate tumor models, animals were treated with intravenous injection of 0.87 mg/kg of PLMDs, and tumors were irradiated with 10 Gy 4 h later. At 5 d post-treatment, the tumors were resected and stained for PD-L1 expression. The results demonstrated that saline-treated animals exhibited very low levels of PD-L1 expression in PC3 tumors (Fig. 3H and I), while PD-L1 expression in TRAMP-C2 tumors was 10-fold higher than that in PC3 tumors (Fig. 3H to J). PLMD treatment alone reduced the expression of PD-L1 in TRAMP-C2 tumors. RT alone showed an increase in PD-L1 expression by 20-fold in PC3 and 1.5-fold increase in TRAMP-C2 tumors compared to saline groups. PLMDs plus RT treatment significantly reduced PD-L1 expression by approximately 5-fold compared to RT alone in PC3 and TRAMP-C2 tumor models (Fig. 3H). These results demonstrate that for both PC3 and TRAMP-C3 cell lines, PLMDs can down-regulate RT-induced PD-L1 expression in vitro and in vivo.

### Systemic PLMD administration with localized RT boosts antitumor immune responses in a syngeneic TRAMP-C2 tumor model

**Fig. 4.**
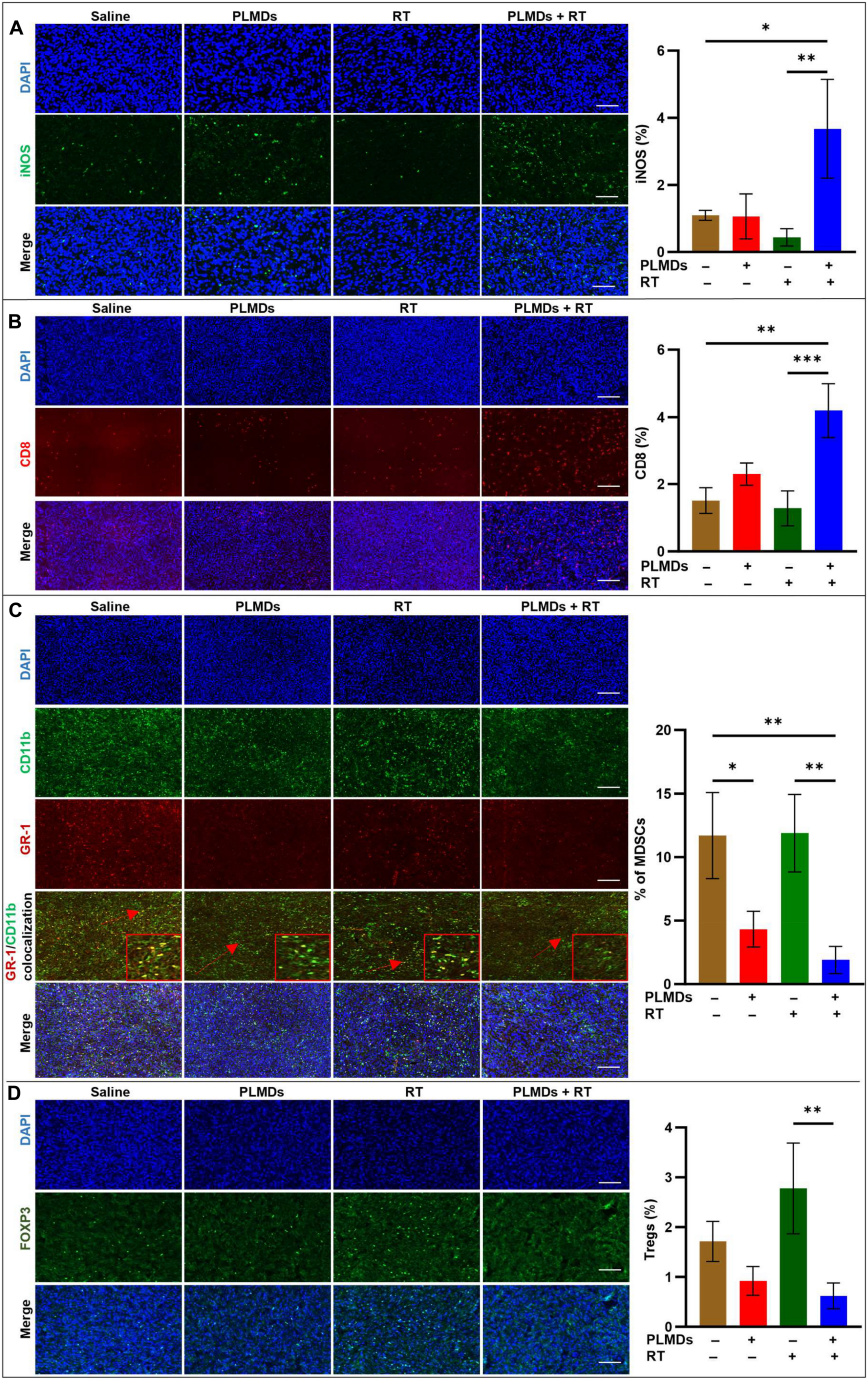
PLMDs plus RT enhanced the infiltration of antitumor immune cells while suppressing the infiltration of tumor-promoting immune cells in TRAMP-C2 tumor tissues. Immunofluorescent images and quantification of iNOS^+^ M1 macrophages (A) and CD8^+^ cytotoxic T cells (B). (C) Immunofluorescence staining of CD11b^+^ and GR-1^+^ colocalization (MDSCs) and (D) FOXP3^+^ (Tregs). Quantification of image data for the indicated markers compared to saline control (*n* = 3), PLMDs alone (*n* = 4), RT alone (*n* = 4), and PLMDs + RT (*n* = 5). Scale bars, 200 μm. Data are presented as means ± SD. *P* < 0.05 (*), *P* < 0.005 (**), *P* < 0.0005 (***), and *P* < 0.0001 (****).

The effect of PLMDs plus localized RT on stimulating antitumor immune responses in vivo was investigated. Animals were treated intravenously with PLMDs (0.87 mg/kg MnO_2_). Four hours later, the tumor was irradiated with a single 10-Gy dose. At 5 d post-treatment, animals were sacrificed, and tumors were removed, fixed, sectioned, and analyzed for the presence of inducible nitric oxide synthase (iNOS^+^) M1 macrophages and CD8^+^ T cells (Fig. 4A and B).

Immunofluorescence staining showed that the percentage of M1 macrophages was significantly increased to 3.5% in PLMDs plus RT-treated tumors compared to 1% in the saline control and PLMDs alone groups, while RT alone slightly decreased iNOS^+^ M1 macrophages compared to the saline control (Fig. 4A). PLMDs slightly increased the infiltration of CD8^+^ T cells compared with saline (Fig. 4B). However, treatment with RT alone exerted no obvious effect on the population of CD8^+^ T cells compared with saline (Fig. 4B). The percentage of CD8^+^ T cells infiltrating into the TRAMP-C2 tumors significantly increased to 4% in the PLMDs + RT group compared to 1.5% in saline group. These results suggest modulation of TME by PLMDs followed by RT enhanced the recruitment of antitumor immune cells to the tumor site.

### Modulation of the TME using PLMDs plus RT suppress recruitment of immunosuppressive cells into tumors

Conditions within the TME have been shown to enhance the recruitment of Tregs and MDSCs to intratumor sites, enhancing immunosuppression and leading to tumor growth [16]. Therefore, we investigated whether PLMDs, alone or in combination with RT, could reduce the recruitment of immunosuppressive Tregs and MDSCs in a murine subcutaneous TRAMP-C2 tumor model in C57BL/6J mice. As shown in Fig. 4C, Gr-1^+^ and CD11b^+^ cells, markers of MDSCs, resided in high numbers in both untreated TRAMP-C2 tumors and RT-treated TRAMP-C2 tumors. Treatment with PLMDs alone significantly reduced the percentage of MDSCs from 12% in the saline group to 4%, whereas RT alone showed no effect in this regard. In contrast, PLMDs plus RT further reduced Gr-1^+^ and CD11b^+^ MDSCs cells to 2%, an approximately 6-fold reduction compared to the saline-treated group (Fig. 4C). Additionally, FOXP3 expression was used as a marker of Tregs in this specific environment. As shown in Fig. 4D, RT increased the density of Tregs compared to that observed in the saline controls. Pretreatment of TRAMP-C2 tumors with PLMDs followed by RT resulted in a 2.5-fold reduction in Tregs compared with RT alone (Fig. 4D). PLMDs alone also reduced the recruitment of Tregs compared to that in the saline group (Fig. 4D). These results indicate that TME modulation by PLMDs inhibited the infiltration of immunosuppressive cells to the tumor site.

### PLMDs enhance the antitumor effect of RT in TRAMP-C2 tumor-bearing mice

**Fig. 5.**
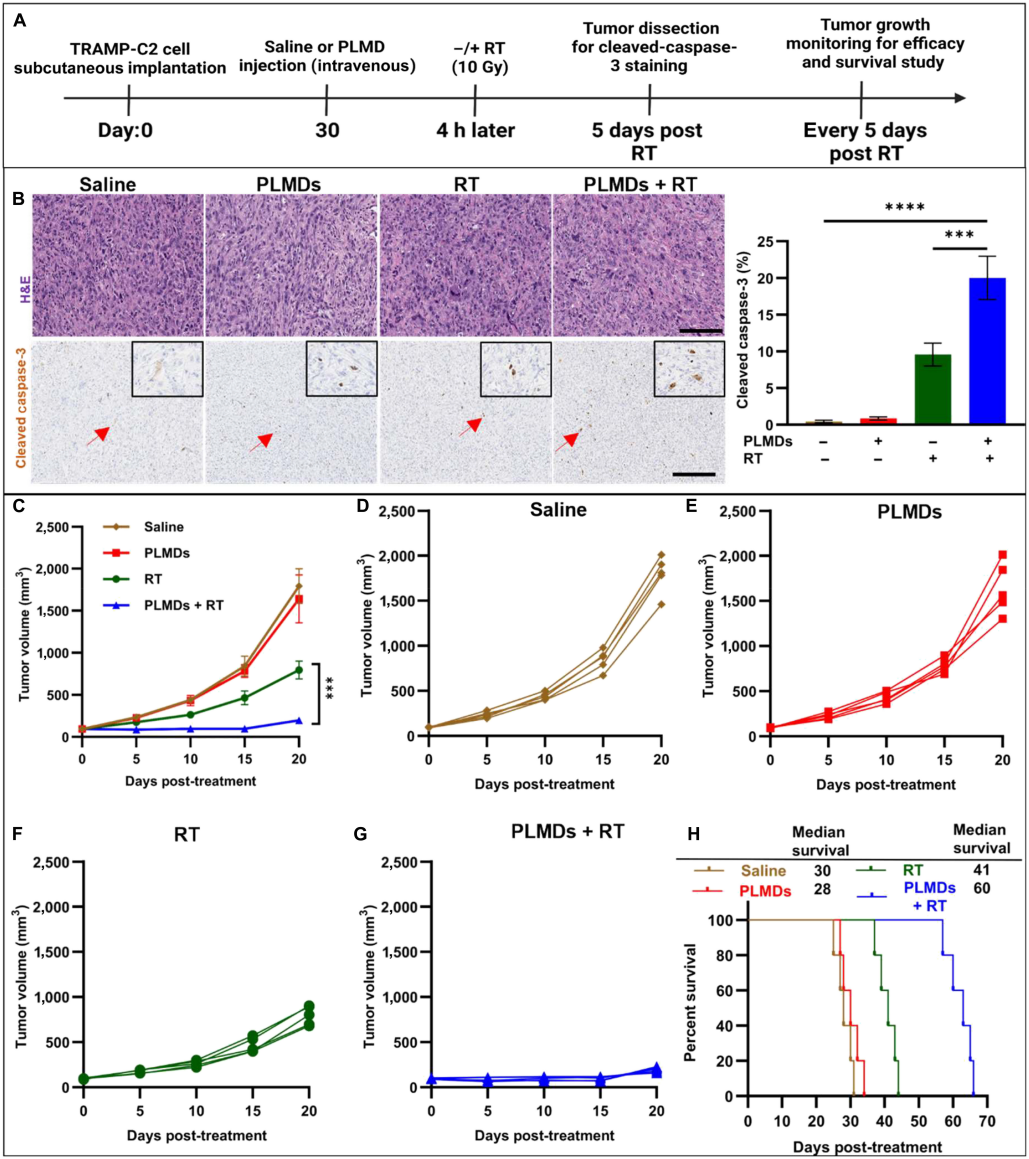
PLMDs plus RT induces apoptosis and inhibits tumor growth and prolonged median survival of TRAMP-C2 tumor-bearing mice. (A) Schematic of PLMDs and RT treatments of subcutaneous flank TRAMP-C2 tumors grown in C57BL/6J mice. (B) Tumor tissue stained by hematoxylin / eosin (H&E) and immunohistochemistry for cleaved caspase-3 in TRAMP-C2 tumors 5 d after various treatments. Scale bars, 200 μm. (C) Composite growth curves for the 4 treatment groups. Individual mouse tumor growth curves for (D) saline group, (E) PLMDs group animals, (F) RT alone-treated animals, and (G) PLMDs + RT-treated animals. (H) Kaplan–Meier survival curves for TRAMP-C2 tumor-bearing mice receiving various treatments. Tumor growth kinetics of following treatment with saline (*n* = 5), PLMDs (0.87 mg MnO_2_/kg bw) (*n* = 5), RT alone (10 Gy) (*n* = 5), and PLMDs + RT treatment (*n* = 5). The numbers in the plot indicate the median survival days for each treatment group. RT (10 Gy) was locally delivered 4 h post intravenous injection of PLMDs. Data are expressed as means ± SD. *P* < 0.05 (*), *P* < 0.005 (**), *P* < 0.0005 (***), and *P* < 0.0001 (****).

Given the enhanced infiltration of CD8^+^ T cells in tumors along with enhanced radiation efficacy in vitro, we were interested in whether such effects would translate to an enhancement in cell death within tumors. Therefore, we examined tumor sections for the expression of activated caspase-3, a marker of apoptosis [45]. TRAMP-C2 tumor-bearing mice were treated with intravenous injected PLMDs, and the tumors were irradiated 4 h later (Fig. 5A). As shown in Fig. 5B, at day 5, PLMDs plus RT treatment induced a significant increase in activated caspase-3 expression in the tumors compared to saline controls. These results indicate that PLMDs + RT substantially exacerbated the levels of induced cellular apoptosis compared to RT or PLMDs alone. To further assess the functional antitumor efficacy of PLMDs plus RT, the animals were monitored, and the tumor volumes were measured every 5 d post-RT. While PLMDs alone did not delay tumor growth and increase the survival compared to saline control, RT (10 Gy) delayed tumor growth and prolonged the median survival time by 42% compared to saline group (Fig. 5C, F, and H). More significantly, PLMDs plus RT profoundly suppressed tumor growth and increased median survival by 121% compared to saline and by 55% compared to RT alone (Fig. 5C, G, and H). These results suggest that TME modulation using PLMDs enhances the antitumor effect of RT against TRAMP-C2 tumors.

## Discussion

**Fig. 6.**
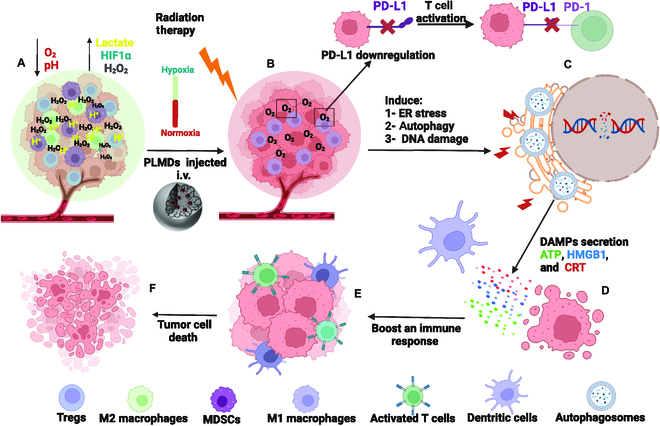
Proposed mechanism of antitumor effects of PLMDs plus RT. Schematic illustration shows tumor before (A) and after reoxygenation (B), reducing acidosis and ROS by PLMDs [30] resulting in PD-L1 down-regulation (B). PLMDs plus RT activated autophagy, ER stress, and enhanced DNA damage (C), inducing the release of DAMPs (D). In addition, remodeling of the immune tumor microenvironment using PLMDs plus RT reduced MDSCs and Tregs (E) while increasing the conversion of M2 to M1 macrophages (E) and increased infiltration of CTL in tumors (E), leading to apoptosis (F). PD-1, programmed death-1.

Stimulating the host immune response against cancer cells using various therapeutic approaches, such as immunotherapy, has been employed with increasingly positive results in solid tumors but has yet to be demonstrated in PCa [46]. In part, this is because PCa is considered a “cold” tumor, characterized by low PD-L1 expression, making it unsuitable for treatment using checkpoint inhibitors. Furthermore, infiltration of immunosuppressor cells, for example, Tregs and MSDCs, lack CTL infiltration, and hostile TME conditions such as hypoxia, acidosis, and ROS act to aid the immune escape of PCa cells [36,47,48]. Although standard cancer therapies, such as RT, can induce an ICD-mediated antitumor immune response via stimulating tumor cells to secrete DAMPs [49], this response may be inadequate to trigger effective antitumor immunity due to inefficient autophagy or apoptotic induction, tumor hypoxia, and radiation-induced PD-L1 up-regulation [50,51]. Previously, we have demostrated the ability of PLMDs to modulate TME through reoxygenating the tumor, down-regulating hypoxia-inducible factor 1α, increasing tumor pH, and reducing ROS [30,31,34]. In this study, we demonstrated that modulation of the TME using PLMDs followed by RT significantly enhanced RT-induced DNA damage, inducing autophagy and ER stress, and DAMP release. Additionally, while RT alone up-regulated PD-L1 expression, combinatorial treatment with PLMDs and RT resulted in a significant reduction in both PD-L1 expression and recruitment of immunosuppressive cells such as MDSCs and Tregs. Furthermore, treatment with PLMDs + RT enhanced the accumulation of immunostimulatory cells, such as M1 macrophages and cytotoxic T cells, leading to greater apoptosis and tumor growth inhibition (Fig. 6).

In autophagy-competent human PC3 and murine TRAMP-C2 PCa cells, PLMDs alone or PLMDs with RT increased autophagy, whereas in autophagy-impaired DU145 cells, the same treatments did not show any effect. In agreement with the literature, ATP release was only observed in the autophagy-competent cell lines PC3 and TRAMP-C2 [9,10].

Our data showed that PLMDs induced the polarization of tumor-associated macrophage into the M1 phenotype and reduced the density of MDSCs and Tregs recruited into the tumor, which reportedly inhibits the antitumor function of T cells [52]. We found that the reduction in MDSC recruitment in tumor treated with PLMDs plus RT was associated with increased T cell infiltration and induced apoptosis, resulting in slower tumor growth and prolonged median survival. These results suggest that the hypoxia-diminishing and ROS-scavenging effects of PLMDs modulate the immunosuppressive environment, leading to the activation of antitumor immune cells when combined with RT.

Although the in vivo study showed that PLMDs alone did not inhibit tumor growth, they modulated the TIME, enhanced the efficacy of RT, and boosted RT-induced antitumor immunity, suggesting that PLMDs plus RT has the potential to convert cold tumors into hot tumors and improve the outcome of RT alone or in combination with T cell-based immunotherapy in cold tumors like CRPC.

In conclusion, we demonstrated that modulation of the TME using oxygen-generating PLMDs in conjunction with RT substantially potentiated immune activation against tumors, as evidenced by significant induction of ICD, autophagy, and ER stress, as well as lethal enhancement of DNA damage. PLMDs induced alterations in the TIME by stimulating the conversion of macrophages toward an antitumor M1 phenotype. Most importantly, the combination of PLMDs and RT significantly inhibited the recruitment of immunosuppressive MDSCs and Tregs while increasing the proportion of antitumor infiltrating CD8^+^ T cells to tumors in vivo. Collectively, TIME remodeling and RT potentiation by PLMDs led to a significant induction of apoptosis, resulting in tumor growth inhibition. These novel hypoxia-attenuating and radiation-sensitizing PLMDs in combination with RT may exhibit advantages over checkpoint inhibitors with respect to boosting antitumor immunity in cold tumors, such as CRPC, via multiple pathways. This new treatment modality may be exploited to enhance T cell-based immunotherapy by generating T cell friendly TME.

## Materials and Methods

### Cell lines and reagents

PC3 cells (derived from Grade IV human prostate adenocarcinoma), DU145 cells (derived from CNS metastasis of PCa patients), and murine TRAMP-C2 cells (derived from prostate adenocarcinoma in a C57BL/6 mouse) were obtained from American Type Culture Collection (Manassas, VA, USA) and used in this study as a PCa model. PC3 and DU145 cells were grown in α-minimal essential medium (Thermo Fisher Scientific, Waltham, MA, USA) supplemented with 10% fetal bovine serum (Thermo Fisher Scientific, Waltham, MA, USA). TRAMP-C2 cells were grown in Dulbecco's modified Eagle's medium supplemented with 4.5 g/l glucose, 0.005 mg/ml and 10 nM dehydroisoandrosterone, 5% fetal bovine serum and 5% Nu-Serum IV (Sigma-Aldrich, Missouri, USA). Unless otherwise indicated, all cell lines were grown in a humidified incubator (NuAire, Plymouth, MN, USA) at 37 °C and 5% CO_2_ (Linde, Mississauga, Ontario, Canada). For in vitro hypoxic experiments, cells were grown in a humidified incubator (New Brunswick Galaxy 14S, Eppendorf, Mississauga, Ontario, Canada) with a gas mixture of 1% O_2_ [37], 5% CO_2_, and 94% N_2_ gas (Linde, Mississauga, Ontario, Canada) at 37 °C. Antibodies against p62/SQSTM1, PD-L1, γ-H2AX, anti-CD11b, CRT, β-actin, and anti-Gr-1 were purchased from Abcam (Cambridge, UK). Antibodies against CD163, CD86, microtubule-associated protein 1A/1B-light chain 3 (LC3), and iNOS were purchased from Novus Biologicals (Littleton, CO, USA). Antibodies against CCAAT-enhancer-binding protein homologous protein (CHOP), caspase-3, PD-L1, cleaved caspase-3, and CD8a were purchased from Cell Signaling Technology (Danvers, MA, USA).

### In vitro treatment and immunoblotting

PC3, DU145, or TRAMP-C2 cells (1 × 10^5^) were plated onto 6-cm plastic dishes (Thermo Fisher Scientific Inc., USA) containing 3 ml of growth medium and grown under hypoxic conditions at 37 °C for 48 h. Hypoxic PC3, DU145, or TRAMP-C2 cells were incubated with PLMDs (12.5 μMMnO_2_) for 1 h at 37 °C and then irradiated with a single 6-Gy dose (X-RAD 320, Precision X-Ray, North Branford, CT, USA). The energy/tube current of the x-rays was 320 kV/12.5 mA, and the dose rate was 2.7 Gy/min. At 4 and 24 h post-treatment, cell containing dishes were washed with cold phosphate-buffered saline (PBS), and cells collected in sodium dodecyl sulfate polyacrylamide gel electrophoresis (SDS-PAGE) reducing buffer (2% SDS, 62.5 mM tris-HCl pH 6.8, 10% glycerol, 0.05% bromophenol blue, and 5% β-mercaptoethanol). Cell lysates were sonicated (75HT Ultrasonic Cleaner, VWR International LLC., Mississauga, ON, CA) at 60 Hz for 20 s, followed by heating to 95 °C for 5 min, electrophoresed with 4% to 15% SDS-PAGE (Bio-Rad, Hercules, CA, USA), and then transferred to nitrocellulose membrane using the Trans-Blot Turbo Transfer System (Bio-Rad, Hercules, CA, USA). The membranes were then incubated in 5% of bovine serum albumin (BSA) blocking buffer for 1 h at room temperature. Primary antibodies against LC3 and CHOP were diluted 1:1,000 in blocking buffer to specific concentrations and incubated with the nitrocellulose membrane for 1 h at room temperature, followed by incubation with horseradish peroxidase-conjugated secondary antibodies at a dilution of 1:15,000 in blocking buffer for 1 h. Protein quantification was performed relative to that of β-actin. Images were captured using a ChemiDoc MP Imaging System (Bio-Rad, Hercules, CA, USA) and analyzed using Image Lab Software (Bio-Rad, Hercules, CA, USA).

### Investigation of ICD markers in human and murine PCa cells

Cancer cells were seeded in 96-well plates (5 × 10^4^ cells per well) and cultured under hypoxic conditions for 48 h. The cells were then treated with saline, PLMDs (12.5 μM MnO_2_), RT (6 Gy), or PLMDs for 1 h followed by RT (6 Gy). After further 24 h of incubation under hypoxic conditions at 37 °C, cell supernatants were collected, and the release of HMGB1 and ATP detected using HMGB1 ELISA Kit (Tecan Group LTD, Switzerland) and Chemiluminescence ATP Determination Kit (Sigma-Aldrich, Missouri USA), according to the manufacturer’s protocols respectively. The translocation of CRT was assessed by CLSM. Cells were seeded on glass coverslips in 6-well plates at a density of 1 × 10^5^ cells per well and cultured for 48 h under hypoxic conditions at 37 °C. The cells were then treated with saline, PLMDs (12.5 μM MnO_2_), RT (6 Gy), or PLMDs + RT. One hour after the treatment, the cells were irradiated with a single dose of 6 Gy and kept in the same gas atmosphere and temperature as prior to radiation. After 4 h, the cells were washed 3 times with cold PBS, fixed with 4% paraformaldehyde for 10 min, washed, and blocked with 1% BSA for 30 min at room temperature. Cells were then incubated with CRT antibody (1:200) for 1 h at room temperature, washed and incubated with Alexa Fluor 488 anti-rabbit secondary antibody (1:10,000) for 1 h at room temperature, and stained with 4′,6-diamidino-2-phenylindole at room temperature for 5 min. Immunofluorescence images were obtained using a Zeiss Axio Scan Z1 slide scanner (Zeiss, Oberkochen, Germany).

### Macrophage polarization study and Transwell assay for cocultured RAW264.7 macrophages and TRAMP-C2 cells

For macrophage polarization, RAW264.7 macrophages (0.5 × 10^5^ cells) in 3 ml of growth media were seeded onto 6-well plates and grown under normoxic or hypoxic conditions at 37 °C for 48 h. Cells were then incubated with PLMDs (12.5 μM MnO_2_) for 4 h at 37 °C. Cells were then washed with PBS, stained for M1 and M2 macrophage markers CD86 and CD163 at 1:400 dilution, respectively, and incubated with secondary antibody at 1:1,000 dilution for 1 h at room temperature. The growth media was collected for TNF-α measurement using an enzyme-linked immunosorbent assay kit (Thermo Fisher Scientific Inc., USA) according to the manufacturer’s instructions.

The in vitro antitumor effect of PLMDs-induced RAW264.7 cell polarization on TRAMP-C2 cells was investigated using a Transwell assay. Briefly, 0.5 ×10^5^ of RAW264.7 macrophages were seeded in Transwell inserts (upper, 0.4-μm pores; Greiner Bio-one), allowing their exposure to secreted molecules but not in direct cell–cell contact. Transwells were inserted into a 6-well plate containing 1 × 10^5^ TRAMP-C2 cells (bottom). The entire system was cultured under normoxic or hypoxic conditions for 48 h at 37 °C with 12.5 μM of PLMDs added to inserts. After 24 h, the TRAMP-C2 cells were washed with cold PBS and stained with crystal violet. The relative levels of cell viability were then assessed using light microscopy to detect groups of adherent TRAMP-C2 cells.

### Immunofluorescence microscopy for biomarker detection

Cells cultured on sterile glass coverslips (0.15-mm thickness) in the indicated growth media were washed with cold PBS and fixed with 4% paraformaldehyde for 10 min at room temperature. Cells were then permeabilized using 0.5% Triton X-100 for 10 min at 4 °C and incubated with 1% BSA blocking buffer for 1 h at room temperature. Primary antibodies were incubated for 1 h at room temperature, following the manufacturer’s instructions, followed by washing with blocking buffer 3 times for 5 min per wash. The cells were incubated with secondary antibodies for 1 h at room temperature according to the manufacturer’s instructions. Images were captured using a Zeiss Axio Scan Z1 slide scanner (Zeiss, Oberkochen, Germany). Images were analyzed using the HALO software (Indica Labs, Albuquerque, NM, USA).

### In vivo tumor studies

All procedures were conducted in accordance with the ethical and legal requirements of the Animals for Research Act of Ontario and the Federal Canadian Council on Animal Care Guidelines for the Care and Use of Laboratory Animals. The animal protocols used in these studies were approved by the University of Toronto Animal Care Committee and/or University Health Network Animal Care Committee (Animal Use Protocol 6383). PC3 tumors were grown in 8- to 10-week-old male severe combined immunodeficiency mice (Jackson Laboratories, Bar Harbor, ME, USA). TRAMP-C2 tumors were grown in C57BL/6 mice (Ontario Cancer Institute, Toronto, Ontario, Canada). TRAMP-C2 cells (3 × 10^6^) or PC3 cells (1.0 × 10^6^) in a 1:1 mixture of growth medium plus 10% Matrigel (Corning, Corning, NY, USA) were injected in a volume of 30 μl subcutaneously into the right flank under isoflurane inhalational anesthesia. Once tumors reached 100 mm^3^, animals were randomly assigned to 1 of 4 treatment groups: (a) Saline; (b) PLMDs alone (1 mM MnO_2_, 0.87 mg MnO_2_/kg body weight [bw]); (iii) RT alone (10 Gy); or (iv) PLMDs + RT. RT was administered 4 h after PLMDs injection. Tumors were then irradiated using an XRAD 225Cx irradiator with photon energy of 225 KVp at 2.56 Gy/min and a tube current of 13-mA and 0.3-mm copper filter. The 4-h time interval after PLMD intravenous injection and RT dose were selected based on our previous studies [30–32,34]. Five days after radiotherapy, mice were sacrificed by cervical dislocation under 1% isoflurane anesthesia. Tumors were immediately removed and fixed in 10% neutral-buffered formalin, paraffin-embedded, sectioned, and stained with the antibodies indicated. For antitumor efficacy studies, tumor size was measured using a Vernier caliper and tumor volume (*V*) was calculated using the following formula:

$$V=\frac{\left(\text{length}\right)\times {\left(\text{width}\right)}^{2}}{2}$$

### Statistical analysis

Statistical analyses were performed using GraphPad 1-way ANOVA and Tukey’s post hoc test. Error bars represent means ± SD. Probability (*P*) values less than 0.05 (*P* < 0.05) were considered statistically significant and are designated as *P* < 0.05 (*), *P* < 0.005 (**), *P* < 0.0005 (***), and *P* < 0.0001 (****).

## Data Availability

The data generated in this study are available in the article and supplementary data or upon request from the corresponding author.

## Supplementary Materials

Supplementary 1 Synthesis and characterization of PLMDs. Figs. S1 and S2
