# Supplementary material for: Remodeling Tumor Immune Microenvironment by Using Polymer-Lipid-Manganese Dioxide Nanoparticles with Radiation Therapy to Boost Immune Response of Castration-Resistant Prostate Cancer
Source: Research (Wash D C). 2023 Oct 3;6:0247. doi: 10.34133/research.0247 (PMC10546607; doi:10.34133/research.0247)
Supplement: Supplementary 1 — Synthesis and characterization of PLMDs. Figs. S1 and S2 [file research.0247.f1.docx]

**Supplementary materials**

Synthesis and characterization of PLMDs

PLMD were prepared following our previous protocol (30-34) with minor modifications The detailed method was reported in the Supplementary Information in ref. 33. Briefly, an aqueous solution of manganese permanganate (KMnO4) was first reduced with the action of poly(allylamine hydrochloride) (PAH; Sigma-Aldrich, St. Louis, MO , USA) to produce MnO_2_ nanoparticles (MD NPs) (~10 nm diameter). Oleic acid (Sigma-Aldrich) was added to form a covalent bond with the hydrophilic MD NPs precursor and transfer them into an organic solvent. Finally, MD NPs were added into solid polymer-lipid nanoparticles of myristic acid in the presence of polyoxyethylene (40) stearate and polyoxyethylene (100) stearate using an oil-in-water emulsion method, resulting in PLMDs formation. The physicochemical properties of PLMDs were characterized following the protocol published in our previous studies (30-32). Briefly, the particle size, particle size distribution, and zeta potential of the PLMD were measured by dynamic light scattering (DLS, Zetasizer- Malvern), and the morphology of MDNPs was confirmed using transmission electron microscope.


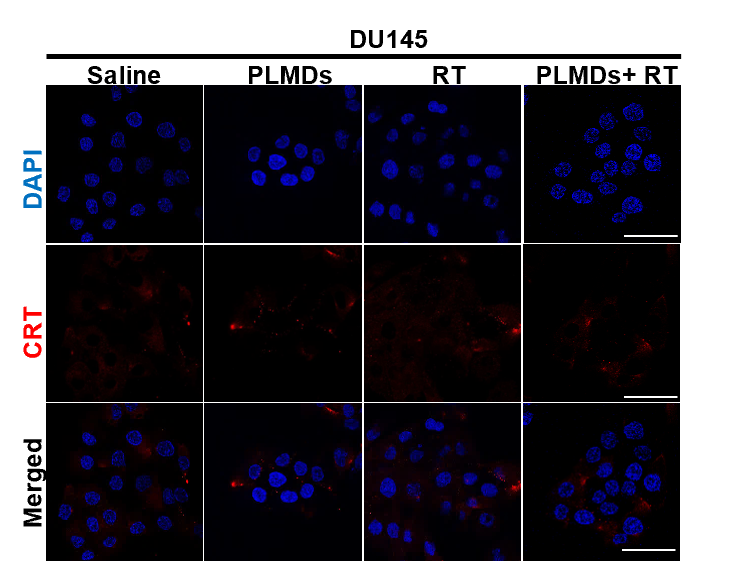


**Figure S1**. Confocal microscope images of CRT exposure in hypoxic DU145 cells *in vitro*. DU145 cells were treated with saline, PLMDs (12.5 µM), RT (6 Gy) or PLMDs plus RT under hypoxic conditions and CRT exposure was assessed 4 h post-treatment. Scale bars, 20 µm.


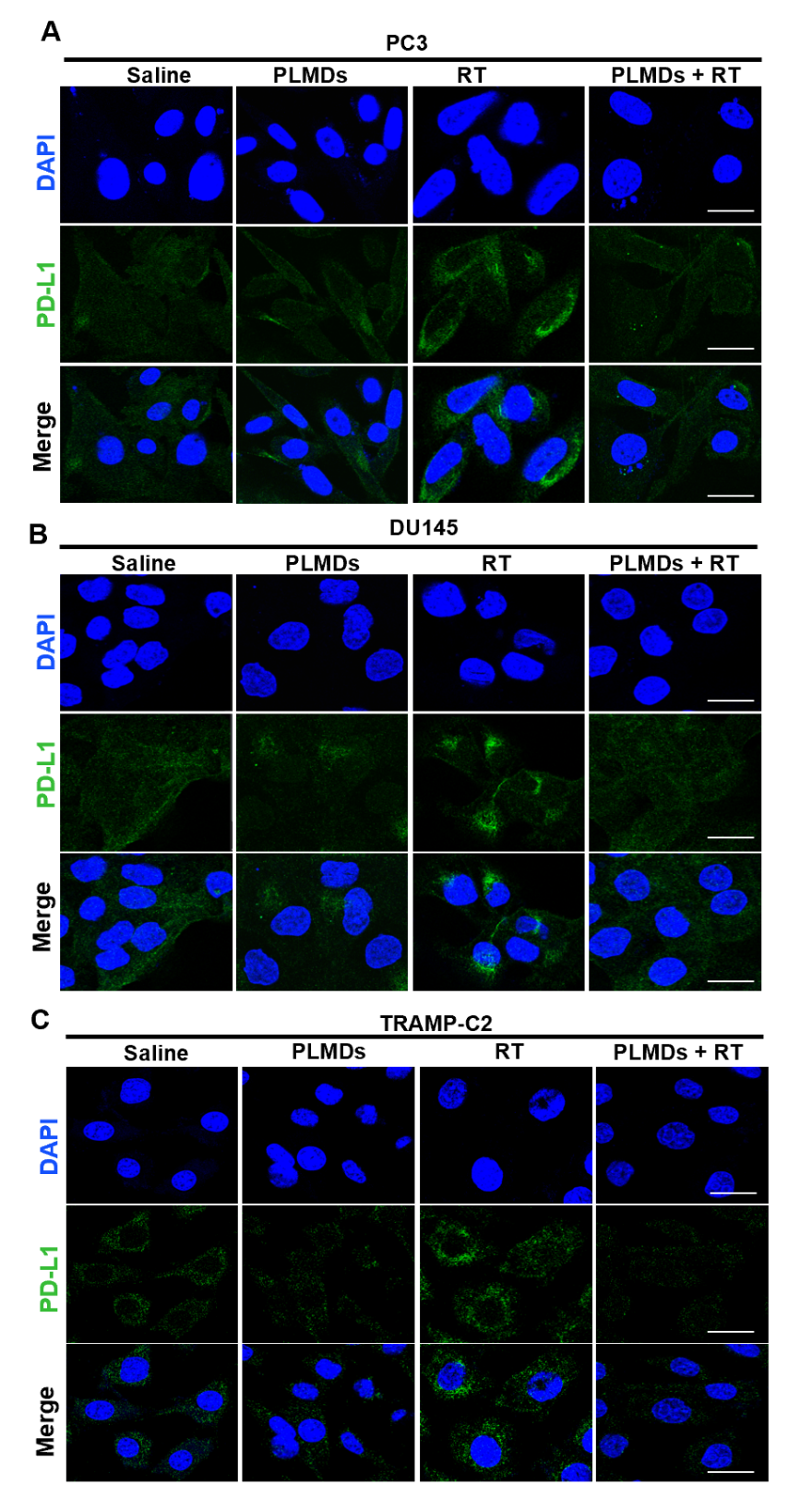


**Figure S2**. Confocal microscope images of PDL-1 expression in hypoxic PC3 (A), DU145 cells (B) and TRAMP-C2 (C) cells *in vitro*. Cells were treated with saline, PLMDs (12.5 µM), RT (6 Gy) or PLMDs plus RT under hypoxic conditions for 48 h then PD-L1 expression was assessed 24 h post-treatment. Scale bars, 10 µm.
